# Exposure of the Brazilian population to risk factors for oral cancer

**DOI:** 10.1590/1807-3107bor-2025.vol39.105

**Published:** 2025-10-10

**Authors:** Hannah Gil de Farias MORAIS, Lucas Melo da COSTA, Renata Roque RIBEIRO, Maria Helena Rodrigues GALVÃO, Angelo Giuseppe RONCALLI, Márcia Cristina da Costa MIGUEL, Roseana de Almeida FREITAS

**Affiliations:** (a) Universidade Federal do Rio Grande do Norte - UFRN, School of Dentistry, Postgraduate Program in Dental Sciences, Natal, RN, Brazil.; (b) Universidade Federal de Pernambuco – UFPE, Academic Center of Vitória, Vitória de Santo Antão, PE, Brazil.

**Keywords:** Mouth Neoplasms, Carcinoma, Squamous Cell

## Abstract

The aim of this study was to investigate the profile of the Brazilian population exposed to risk factors for oral cancer, as well as the profile of patients in the high-risk group for this disease in Brazil. This study relied on a cross-sectional design, utilizing secondary data from the 2020 National Health Survey (PNS). The database used contained information on 90,846 individuals aged 18 and older. The dependent variable was “High-risk group for oral cancer development” and the independent variables were grouped into biological characteristics, educational level, access to health services, and self-care practices. In the multivariate analysis, odds ratios of outcomes were assessed using a logistic regression model. Individuals of white ethnicity, with low levels of education, residing in rural areas, not registered in the Family Health Strategy program, who have rarely or never seen a dentist, self-assessing their general health as poor, and lacking regular physical exercise were more likely to be in the high-risk group for the development of oral cancer. The data from the 2020 National Health Survey (PNS) demonstrate that a large portion of the Brazilian population is exposed to risk factors for oral cancer. This highlights the disparities in access to medical and dental health services, as well as in the educational and preventive interventions by healthcare professionals regarding risk factors for oral cancer.

## Introduction

In the international scenario, the incidence and mortality of oral cancer rank sixteenth among all malignancies,^1,2^ and in Brazil, cancer is the third leading cause death in the population, with oral cancer holding a prominent position, resulting in the death of over six thousand Brazilians annually.^3,4^ Oral squamous cell carcinoma (OSCC) is the most prevalent malignant oral neoplasm, predominantly affecting men around the sixth decade of life, representing the main risk group for OSCC development.^5^

In addition to the epidemiological profile, tobacco (or its derivatives) and alcohol consumption are the main risk factors associated with oral carcinogenesis, while sun exposure is linked to lower lip SCC and human papillomavirus (HPV) infection to oropharyngeal lesions.^6,7^ Understanding other parameters such as dietary consumption patterns, overweight status, and individual lifestyle can also be useful in assessing the health status of patients, as these characteristics are considered contributing factors to oral carcinogenesis.^8^ Socioeconomic and educational levels and access to healthcare services can also help delineate the epidemiological landscape of SCC, considering that these factors influence disease prognosis and mortality,^9,10^ and may also contribute to delayed diagnosis, leading to higher treatment costs.^11^

Therefore, understanding population habits can help to better comprehend the community in which the healthcare professional operates and identify high-risk patients, aiding in the early detection of malignant oral lesions.^1^ Thus, it is important to investigate the profile of the Brazilian population in relation to OSCC concerning sociodemographic parameters, access to healthcare services, habits, and lifestyle. The aims is to understand how these factors are present and interact within our society to outline a risk profile specific to the Brazilian population. This will facilitate screening and the adoption of public policies, with potential benefits for the early detection of malignant oral lesions, improved prognosis, and especially prevention of this condition in the population.

## Methods

### Design and participants

This was a cross-sectional study using secondary data from the National Health Survey (PNS) conducted between 2019 and 2020, which is a population-based household survey with national coverage, conducted by the Brazilian Institute of Geography and Statistics (IBGE) in partnership with the Ministry of Health. This survey aims to understand the determinants, conditions, and health needs of the Brazilian population. The data are representative of the country and the population residing in private households.^12^

The database used contains information on 108,457 households, of which 100,541 were occupied. Of the occupied households, 94,114 household interviews were conducted, resulting in a response database for 279,382 individuals. After excluding refusals, residents that were not found, and ignored data, 90,846 interviews remained, which comprised the sample of this study.^12,13^

### Study variables

In order to understand the profile of the Brazilian population, sociodemographic information, access to healthcare services, health investigation, and habits/lifestyle data were collected. Table 1 presents the original and derived variables used in this study.

**Table 1:** Re-categorization of variables from the PNS (2020) for the assessments conducted.

Original variables (PNS, 2020)			Re-categorization
Variable code	Description	Categories	Categories
C006	Sex	Man	Feminine
		Woman	Masculine
		Not applicable	
C008	Age of the resident on the reference date	000 a 130 years	N.R.
C009	Skin color or race	White	White
		Black	Black
		Yellow	Brown
		Brown	
		Indigenous	
		Ignored	
		Not applicable	
V0026	Type of census situation	Urban	N.R.
		Rural	
VDD004A	Highest level of education achieved (individuals aged 5 years and older) standardized for Elementary Education - 9-year system	No instruction	No instruction
		Incomplete elementary education or equivalent	Elementary
		Completed elementary education or equivalent	High school
		Incomplete high school education or equivalent	Graduate
		Completed high school education or equivalent	
		Incomplete higher education or equivalent	
		Completed higher education	
		Not applicable	
A01901	Does any resident have access to the Internet at home through a computer, tablet, mobile phone, television, or other device?	Yes	Yes
		No	No
		Ignored	
		Not applicable	
VDF003	Per capita household income (including the income of individuals whose status in the household unit was pensioner, domestic worker, or relative of the domestic worker)	00000000 to 99999999 real	N.R.
N001	Overall, how would you rate your health?	Very good	Good or very good
		Good	Regular
		Regular	Poor or very poor
		Poor	
		Very poor	
		Ignored	
		Not applicable	
U005	Overall, how would you rate your oral health (teeth and gums)?	Very good	Good or very good
		Good	Regular
		Regular	Poor or very poor
		Poor	
		Very poor	
		Ignored	
		Not applicable	
B001	Is your household registered with the family health unit?	Yes	Yes
		No	No
		Don’t know	Don’t know
		Ignored	
I00101	Do you have any private dental plan, company-sponsored plan, or public agency plan?	Yes	Yes
		No	No
		Ignored	
		Not applicable	
I00102	Do you have any private medical insurance plan, company-sponsored plan, or public agency plan?	Yes	Yes
		No	No
		Ignored	
		Not applicable	

The second stage consisted of selecting the dependent variable for this study, which involved determining the “High-risk group for oral cancer development”. The variables considered were selected based on the main oral cancer risk factors already recommended and established according to the World Health Organization (2022). For this purpose, the following characteristics were grouped: men aged 60 years or older, who use any tobacco product and consume alcoholic beverages, smoke, or work outdoors and are exposed to the sun.

For this stage, the independent variables used in the first stage answered by all interviewees were selected, excluding those with previous filters. These were grouped into biological characteristics, educational level, access to healthcare services, and self-care practices.

### Statistical analysis

For statistical analysis, the svy package (for complex sample analysis) of STATA software version 14.2 was used. Descriptive statistical analyses were performed using relative frequency, with expansion to the Brazilian population in the year of the study. The expansion to the Brazilian population was achieved through complex sampling. Descriptive analysis of the data used weighted estimates of the sampling units in the three stages. Each observation incorporated its sampling weight to calculate the estimates and their respective confidence intervals. Thus, the data were described using prevalence estimates and their 95% confidence intervals (95% CI) for the Brazilian population. Bivariate logistic regression analysis was conducted to obtain odds ratios for outcomes between groups. Subsequently, multivariate analysis was also performed using a logistic regression model. To assess the goodness of fit of the model, we applied the Hosmer-Lemeshow test. Variables with a p-value < 0.2 were included in the initial modeling, and in the final model, variables with a p-value < 0.05 were retained, considering a 5% confidence level.

## Results

Table 2 presents the sociodemographic characteristics of the study population. The majority considered their overall health and oral health as good or very good (68.3% and 70.2%, respectively), were registered in the Family Health Strategy (ESF) program (61.8%), and did not have private medical (73.4%) or dental (86.9%) insurance. Most of the population (79.9%) visited a doctor at least once a year, compared to 49.6% who visit a dentist within a year. It was also evidenced that 6.8% of the Brazilian population visited a doctor more than 3 years ago or never, while 25.2% never visited a dental office or visited one more than 3 years ago. Dental treatment was the most reported reason for the last consultation (83.6%) (Table 3).

**Table 2:** Number of observations, estimates, and respective 95% confidence intervals of prevalence for the sociodemographic variables used in the study. Brazil, 2020.

Census situation	n _unweighted_	n _weighted_	%		95%CI
Gender					
Female	48.047	89.179.594	52.9		52.4–53.5
Male	42.799	79.246.594	47.1		46.5–47.6
Skin color					
White	33.133	72.305.966	43.6		42.8–44.3
Black	10.345	19.194.647	11.6		11.2–12.0
Brown	45.994	74.387.145	44.8		44.2–45.5
Census situation					
Urban	79.873	144.745.331	85.9		85.5–86.3
Rural	20.973	23.680.858	14.1		13.7–14.5
Education					
No instruction	7.658	9.837.918	5.8		5.6–6.1
Elementary	35.785	62.781.916	37.3		36.6–37.9
High school	29.824	62.456.605	37.1		36.5–37.7
Graduate	17.579	33.349.750	19.8		19.1–20.5
Internet access					
Yes	69.407	142.853.778	84.8		84.4–85.2
No	21.439	25.572.410	15.2		14.8–15.6
			Mean	SD	95%CI
Average age (years)	90.846	168.426.189	43.3	0.129	43.1–43.6
Monthly income (family)	90.824	168.375.970	1.600	24.276	1.5–1.6

**Table 3:** Number of observations, estimates, and respective 95% confidence intervals of prevalence for the variables of access to services and health investigation used in the study. Brazil, 2020.

Variables	n _unweighted_	n _weighted_	%	95%CI
General health self-assessment				
Good or very good	57.710	115.561.048	68.6	68.0–69.2
Regular	27.113	43.786.710	26.0	25.5–26.5
Poor or very poor	6.023	9.078.431	5.4	5.2–5.6
Oral health self-assessment				
Good or very good	61.705	118.260.910	70.2	69.6–70.8
Regular	23.919	41.637.248	24.7	24.2–25.2
Poor or very poor	5.222	8.528.031	5.1	4.8–5.3
Household registered in ESF*				
Yes	57.500	104.019.987	61.8	60.7–62.9
No	22.512	45.342.317	26.9	25.9–27.9
Don’t know	10.834	19.063.885	11.3	10.8–11.9
Private health insurance				
Medical				
Yes	20.568	44.718.551	26.6	25.8–27.3
No	70.278	123.707.638	73.4	72.7–74.2
Dental				
Yes	10.726	22.097.488	13.1	12.6–13.6
No	80.120	146.328.701	86.9	86.4–87.4
Frequency of visits to healthcare professionals (years)				
Medical doctor				
<1	71.548	134.459.293	79.9	79.3–80.3
1–2	9.277	16.572.600	9.8	9.5–10.2
2–3	3.228	5.870.387	3.5	3.3–3.7
> 3 or never	6.793	11.523.911	6.8	6.5–7.2
Dentist				
< 1	41.669	83.624.648	49.6	49.0–50.3
1–2	15.075	28.465.295	16.9	16.4–17.4
2–3	7.589	13.834.409	8.2	7.9–8.6
> 3 or never	26.513	42.501.838	25.2	24.7–25.8
Reason for dentist consultation				
Dental treatments	35.574	69.927.839	83.6	81.0–86.2
Periodontal treatments	366	1.022.911	1.2	1.0–1.5
Orthodontic or prosthetic treatments	5.520	12.246.878	14.6	13.6–15.8
Mouth wound	65	136.696	0.2	0.1–.03
Others	144	290.319	0.3	0.3–0.4
Inquiry about smoking				
Yes	7.032	13.593.366	61.2	59.7–62.8
No	3.659	6.092.424	27.4	26.0–28.9
Not in the last 12 months	1.582	2.511.824	11.3	10.3–12.4
Treatment for smoking cessation				
Yes	957	1.627.324	15.6	14.1–17.2
No	4.743	8.819.983	84.4	82.8–85.9
Cancer diagnosis				
Yes	2.316	4.072.613	2.4	2.3–2.6
No	88.530	164.353.575	97.6	97.4–97.7
Oral cancer diagnosis				
Yes	91	218.099	5.4	3.7–7.6
No	2.225	3.854.514	94.6	92.4–96.3
Lung cancer diagnosis				
Yes	78	128.476	3.2	2.2–4.5
No	2.238	3.944.136	96.8	95.5–97.8
Breast cancer diagnosis				
Yes	485	987.651	24.3	21.2–27.3
No	1.831	3.084.962	75.7	72.4–78.8
Prostate cancer diagnosis				
Yes	339	589.192	14.5	12.3–16.9
No	1.977	3.483.420	85.5	83.1–87.7
Other cancer diagnosis				
Yes	207	353.180	8.7	7.1–10.5
No	2.109	3.719.432	91.3	89.5–92.9

*“Estratégia Saúde da Família” (Family Health Strategy in literal translation) is a primary healthcare program implemented by the Brazilian government to provide comprehensive healthcare services, emphasizing preventive care and health promotion within communities.

The majority of the population was questioned about smoking habits during consultations with a healthcare professional (61.2%); however, in 84.4% of cases, when patients tried to quit smoking, there was no counseling from a healthcare professional, including healthcare facilities offering treatment. Additionally, 2.4% of the studied population has received a cancer diagnosis. Among the population diagnosed with cancer, 5.4% reported oral cancer, 3.2% lung cancer, 24.3% breast cancer, 14.5% prostate cancer, and 8.7% another type of cancer (Table 3).

Regarding the lifestyle of the studied population, it was observed that the majority had regular access to carbohydrates (90.3%) and proteins (90.2%), unlike vegetables (57.3%), fruits (59.7%), leafy greens (49.2%), and tubers (36.0%), with a large portion of the population still lacking access. For the population engaged in any occupational activity, 23.5% reported prolonged sun exposure. Among those who consumed alcoholic beverages, 24.3% reported consumption more than twice a week. Regarding tobacco habits, 11.0% consumed tobacco products daily and 52.3% reported being former chronic smokers. Details about the types of cigarettes are shown in Table 4. The majority of the population did not engage in physical exercise in the last 3 months (56.6%), and did not use male or female condoms in the last twelve months during sexual intercourse (59.0%). The average weight of the studied population was 72.49 kg (Table 4).

**Table 4:** Number of observations, estimates, and respective confidence intervals (95%) of prevalence for lifestyle variables used in the study. Brazil, 2020.

Variables	n _unweighted_	n _weighted_	%		95%CI
Nutrition					
Carbohydrates					
Yes	82.127	152.143.665	90.3		89.9– 90.7
No	8.719	16.282.523	9.7		9.3– 10.1
Proteins					
Yes	82.701	151.869.847	90.2		89.8– 90.5
No	8.145	16.556.242	9.8		9.5– 10.2
Leafy greens					
Yes	40.739	82.862.272	49.2		48.6– 49.8
No	50.107	85.563.917	50.8		50.2– 51.4
Tubers					
Yes	33.792	60.568.650	36.0		35.4– 36.6
No	57.054	107.857.538	64.0		63.4– 69.6
Vegetables					
Yes	52.474	96.544.662	57.3		56.7– 57.9
No	38.372	71.881.527	42.7		42.1– 43.3
Fruits					
Yes	53.788	100.578.972	59.7		59.1– 60.3
No	37.058	67.847.217	40.3		39.7– 40.9
High sun exposure					
Yes	13.954	23.284.434	23.5		22.8– 24.3
No	38.878	75.644.569	76.5		75.7– 77.2
**Alcoholic beverage**					
Monthly frequency					
Never	55.430	9.223.590	58.9		58.3– 59.6
Less than 1x	11.093	20.138.075	12.0		11.6– 12.3
1x or more	24.323	49.064.523	29.1		28.5– 29.7
Weekly frequency					
Up to 2x	16.122	32.514.896	75.7		74.2– 77.1
More than 2x	4.984	10.445.948	24.3		22.9– 25.9
			Mean	SD	95%CI
Daily intake	35.416	69.202.599	4.3	0.041	(4.2 – 4.4)
**Smoking**					
Tobacco product consumption					
Weekly	1.186	2.011.916	1.2		1.1– 1.3
Daily	10.200	18.453.875	11.0		10.6– 11.3
Non – smoker	79.460	147.960.407	87.8		87.5– 88.2
Former chronic smoker					
Yes	627	1.052.194	52.3		46.8– 57.7
No	559	959.721	47.7		42.3– 53.2
Manufactured cigarettes					
One or more per day	7.060	14.167.331	69.2		67.6– 70.8
One or more per week	783	1.364.518	6.7		5.8– 7.6
Less than 1x per week	332	446.013	2.2		1.8– 2.6
Less than 1x per month	161	220.945	1.1		0.9– 1.3
Does not consume	3.050	4.266.978	20.8		19.6– 22.2
Hand – rolled cigarettes					
One or more per day	3.519	4.975.476	24.3		22.9– 25.8
One or more per week	337	540.434	2.6		2.1– 3.3
Less than 1x per week	109	187.279	0.9		0.6– 1.3
Less than 1x per month	108	234.939	1.1		0.6– 2.1
Does not consume	7.313	14.527.652	71.0		69.3– 72.6
Spice cigarettes					
One or more per day	54	105.071	0.5		0.3– 0.8
One or more per week	28	92.681	0.5		0.2– .09
Less than 1x per week	24	32.317	0.2		0.1– .03
Less than 1x per month	30	56.488	0.3		0.2– 0.5
Does not consume	11.250	20.179.223	98.6		98.1– 99.0
Pipes					
One or more per day	129	156.573	0.8		0.6– 1.0
One or more per week	15	29.078	0.1		0.1– 0.3
Less than 1x per week	9	10.800	0.1		0.0– 0.2
Less than 1x per month	15	15.987	0.1		0.0– 0.2
Does not consume	11.218	20.253.341	99.0		98.7– 99.2
Cigars					
One or more per day	13	13.885	0.1		0.0– 0.1
One or more per week	7	35.615	0.2		0.1– 0.5
Less than 1x per week	19	65.594	0.3		0.1– 0.9
Less than 1x per month	25	61.221	0.3		0.2– 0.5
Does not consume	11.322	20.289.464	99.1		98.6– 99.5
Hookah					
One or more per day	35	115.687	0.6		0.3– 1.1
One or more per week	86	337.932	1.7		1.1– 2.5
Less than 1x per week	51	137.317	0.7		0.4– 1.1
Less than 1x per month	81	205.343	1.0		0.7– 1.5
Does not consume	11.133	19.669.501	96.1		95.1– 96.9
Smokeless tobacco					
Weekly	210	318.950	0.1		0.1– 0.2
Daily	221	249.527	0.2		0.2– 0.2
Does not consume	90.415	167.857.712	99.7		99.6– 99.7
E – cigarette					
Weekly	276	928.772	0.5		0.4– 0.7
Daily	63	141.336	0.1		0.0– .01
No, but has used before	556	1.673.475	1.0		0.8– 1.1
Never used	89.951	165.682.606	98.4		98.2– 98.6
**Physical exercise**					
Engaged in the last 3 months					
Yes	36.398	73.055.013	43.4		(42.7– 44.1)
No	54.448	95.371.176	56.6		(55.9– 57.3)
Weekly frequency					
Does not practice	1.202	2.694.424	3.7		(3.3– 4.1)
Up to 3x/week	20.736	42.544.094	36.0		(34.6– 37.6)
More than 3x/week	14.460	27.818.491	38.1		(35.9– 40.2)
Sexual activity					
Condom use					
Always	14.788	26.589.056	22.8		(22.1– 23.4)
Sometimes	10.600	19.943.373	17.1		(16.5– 17.7)
Never	36.135	68.981.249	59.0		(58.3– 59.8)
Refused to answer	700	1.327.620	1.1		(1.0– 1.3)
			Mean		SD
Weight (kg)	89.954	166.670.035	72.49		0.140

Table 5 presents the bivariate and multivariate analyses and odds ratios for being in the “Risk group for oral cancer development”. The results of the multivariate analysis revealed that individuals of white race or skin color have a higher chance of being in the risk group (OR=1; p<0.001). Individuals with no education (OR: 3.4; p < 0.001) and those with only elementary education (OR=2.4; p<0.001) have a greater chance of being in the high exposure group. Residents of rural areas (OR: 1.8; p < 0.001) and households not registered in the ESF (OR: 1.3; p = 0.012) have a greater chance of being in the oral cancer risk group, as well as those who have never visited a dentist or have not done so for more than 3 years (OR: 1.7; p < 0.001). Regarding self – care practices, individuals who self – assess their overall health as very good (OR: 1.0; p < 0.001) are at a higher chance of belonging to the risk group, as well as those who have not engaged in physical exercise in the last 3 months (OR: 1.7; p < 0.001). The variables of frequency of doctor visits, self – assessment of oral health, and previous diagnosis of oral cancer lost their significance in the multivariate analysis. Figure provides a representative image of the independent risk and protective variables for the oral cancer risk group with their respective odds ratios.

**Table 5:** Bivariate and multivariate analyses of the risk group according to the studied variables using multinomial logistic regression. Brazil, 2020.

Variables	Risk group		Bivariate analysis		Multivariate analysis	
	No	Yes	OR	p – value	OR	p – value
	(%)	(%)				
Biological characteristics						
Race						
White	98.5 (98.3–98.6)	1.5 (1.3–1.7)	1	–	1	–
Black	98.6 (98.3–98.9)	1.4 (1.1–1.7)	0.9 (0.7–1.1)	0.402	0.7 (0.6–0.9)	0.008
Brown	98.7 (98.5–98.8)	1.3 (1.2–1.5)	0.9 (0.7–1.0)	0.093	0.7 (0.6–0.8)	< 0.001
Educational level						
Education						
No instruction	96.2 (95.6–96.8)	3.8 (3.2–4.4)	5.8 (4.2–8.0)	< 0.001	3.4 (2.4–4.8)	< 0.001
Elementary	97.7 (97.5–97.9)	2.2 (2.0–2.5)	3.4 (2.5–4.6)	< 0.001	2.4 (1.8–3.3)	< 0.001
High school	99.3 (99.1–99.4)	0.7 (0.5–0.9)	1.0 (0.7–1.5)	0.835	0.9 (0.6–1.3)	0.689
Graduate	99.3 (99.1–99.5)	0.7 (0.5–0.9)	1	–	1	–
Access to health services						
Census situation						
Urban	98.2 (98.0–98.4)	1.7 (1.6–1.9)	1	–	1	–
Rural	95.9 (95.5–96.2)	4.1 (3.8–4.5)	2.5 (2.2–2.9)	< 0.001	1.8 (1.5–2.0)	< 0.001
Household registered in ESF*						
Yes	97.8 (97.6–97.9)	2.2 (2.0–2.4)	1	–	1	–
No	98.0 (97.7–98.3)	2.0 (1.7–2.3)	0.9 (0.8–1.1)	0.593	1.3 (1.0–1.6)	0.012
Don’t know	98.3 (98.0–98.6)	1.7 (1.4–2.0)	0.7 (0.6–1.0)	0.027	1.0 (0.7–1.2)	0.797
Frequency of visits to healthcare professionals						
Medical doctor						
Less than 1 year	98.6 (98.5–98.7)	1.4 (1.3–1.5)	1	–	–	–
1 to 2 years	98.5 (98.1–98.8)	1.5 (1.2–1.9)	1.1 (0.9–1.4)	0.450	–	–
2 to 3 years	98.7 (98.1–99.1)	1.3 (0.9–1.9)	0.9 (0.6–1.4)	0.780	–	–
More than 3 years or never	98.0 (97.5–98.3)	2.0 (1.6–2.4)	1.5 (1.2–1.8)	< 0.001	–	–
Dentist						
Less than 1 year	99.0 (98.8–99.1)	1.0 (0.8–1.1)	1	–	1	–
1 to 2 years	99.0 (98.8–99.2)	1.0 (0.8–1.2)	1.0 (0.8–1.3)	0.940	0.9 (0.7–1.2)	0.551
2 to 3 years	98.7 (98.3–99.0)	1.3 (1.0–1.7)	1.3 (1.0–1.8)	0.080	1.1 (0.8–1.5)	0.494
More than 3 years or never	97.3 (97.0–97.5)	2.7 (2.4–3.0)	2.8 (2.3–3.3)	< 0.001	1.7 (1.4–2.1)	< 0.001
Cancer diagnosis						
Yes	94.5 (92.3–96.2)	5.4 (3.8–7.7)	1	–	–	–
No	98.0 (97.9–98.1)	2.0 (1.9–2.1)	0.5 (0.3–0.9)	0.012	–	–
Self – care practices						
Self – assessment of general health						
Good or very good	98.8 (98.7–98.9)	1.2 (1.0–1.3)	1	–	1	–
Regular	97.8 (97.5–98.0)	2.2 (1.9–2.4)	1.9 (1.6–2.2)	< 0.001	1.2 (1.0–1.4)	0.022
Poor or very poor	98.6 (98.2–98.9)	1.4 (1.1–1.8)	1.2 (0.9–1.6)	0.170	0.6 (0.5–0.8)	< 0.001
Self – assessment of oral health						
Good or very good	98.7 (98.6–98.8)	1.3 (1.1–1.4)	1	–	–	–
Regular	98.3 (98.0–98.5)	1.7 (1.5–2.0)	1.4 (1.1–1.6)	< 0.001	–	–
Poor or very poor	97.6 (97.0–98.2)	2.3 (1.8–3.0)	1.8 (1.4–2.4)	< 0.001	–	–
Physical exercise practice in the last 3 months						
Yes	98.9 (98.7–99.1)	1.1 (0.9–1.2)	1	–	1	–
No	97.1 (96.9–97.3)	2.8 (2.6–3.1)	2.5 (2.1–3.0)	< 0.001	1.7 (1.4–2.0)	< 0.001

*“Estratégia Saúde da Família” (Family Health Strategy in literal translation) is a primary healthcare program implemented by the Brazilian government to provide comprehensive healthcare services, emphasizing preventive care and health promotion within communities.

**Figure:**
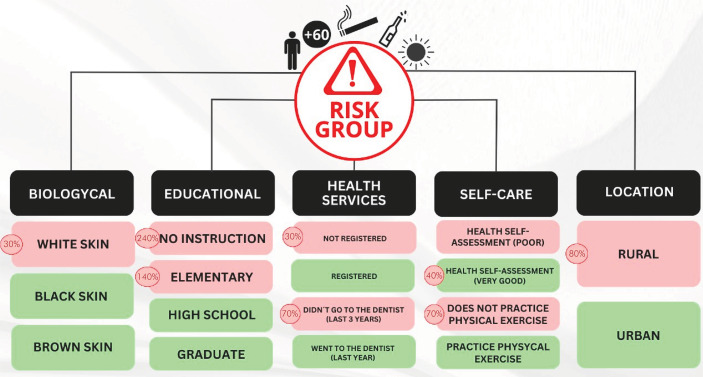
Summary of the main results of this study characterizing the risk group for the development of oral cancer in Brazil, based on biological, educational, access to health services, self – care practices, and housing type, with their risk odds.

The results obtained by the Hosmer – Lemeshow test were F = 1.83 and χ^2^ = 0.119, indicating that the null hypothesis—that the observed and expected frequencies are equal—could not be rejected at the 5% significance level. Therefore, the final model does not present any issues regarding the proposed goodness of fit.

## Discussion

Several studies have identified risk factors for oral cancer, with government – driven educational – preventive initiatives aimed at raising awareness among patients and dentists.^14,15^ However, despite these efforts, oral cancer remains a major public health problem in Brazil and worldwide, with high rates of morbidity and mortality.^16^ In light of this, the identification of high – risk patients for the development of the disease should be emphasized to better guide screening, early diagnosis, and more conservative treatments. With this in mind, we conducted the first nationwide study to identify the portion of the Brazilian population exposed to the main risk factors for oral cancer and the profile of patients belonging to the high – risk group for this disease in Brazil.

There are several risk factors for oral cancer, with smoking standing out as the main cause for neoplastic development,^17^ responsible for about one – third of oral cancer deaths worldwide.^18^ The results of this study revealed that 11% of Brazilians are daily consumers of tobacco products, and more than half of the population (52.3%) are former chronic smokers. Although the risks are known, there is still room for awareness – raising measures in the Brazilian population, with policies aimed at discouraging smoking and its adverse health effects, as well as reinforcing interventions aimed at reducing the still high impact of smoking on the Brazilian population and the global burden of oral cancer.^16^

Alcohol is a well – known risk factor for the development of head and neck cancer, which has been shown to have a synergistic effect with smoking in the tumorigenesis of oral cancer.^19^ The present study revealed that about 40% the Brazilian population consumed alcohol at least once a month, and among drinkers, 24.3% stated that they consume alcohol more than twice a week.

The third risk factor for defining the high – risk group for oral cancer, specifically for the lower lip region, was long – term sun exposure, especially to ultraviolet B radiation, associated with outdoor activities and skin color.^20^ The results revealed a significant portion of the Brazilian population (23.5%) with outdoor occupational activities with prolonged sun exposure. Caution is warranted in interpreting these data, as we cannot estimate lifetime exposure or whether individuals use sun protection. Nevertheless, it is undeniable that many Brazilians are highly exposed to the three main recognized risk factors for oral cancer, including lip cancer.

Dietary habits have been consistently associated with the development of various types of cancer. A pro – inflammatory diet induces persistent inflammation,^21^ promoting the development of cancer in some parts of the body, including oral cancer. However, there are few studies with sufficient detail and quality to establish an association between diet and oral cancer.^22,23,24^ In the current research, we investigated fruit and vegetable consumption, with approximately 40% stating they do not have regular access to these foods. Thus, reducing the inequality in access to healthy food and educating the population on dietary habits may also be an important factor in preventing oral cancer in the Brazilian population.

Considering sexual practices, 59% of Brazilians with sexual activity reported never using condoms. These findings should be considered since unprotected sex exposes individuals to various biological agents, notably the human papillomavirus (HPV), responsible for approximately 5.2% of cancers in humans, including anal, genital, oropharyngeal, and cervical cancers. Although the epidemiology and correlation between HPV infection and oropharyngeal cancer are well – established, many factors remain unknown regarding the association between oral HPV infection and oral cancer.^15^ Despite limited knowledge of the epidemiology, natural history, and prevention of oral HPV infection, studies show that sexually transmitted infections are related to the development of approximately 5% of oral cancer cases.^25^ Although the incidence of HPV – induced oral cancer is low, dentists can educate patients about the risks of unprotected oral sex.

This study also found that individuals of white ethnicity are more likely to be at risk for oral cancer, suggesting that whites have more exposed to carcinogens. However, studies demonstrate that blacks have a higher prevalence of late – stage diagnosis of oral cancer and a higher mortality rate.^26,27^ These findings emphasize racial disparities, with whites being more exposed to risk factors leading to a higher risk of illness, and blacks being diagnosed late, leading to a higher number of deaths.

Education and self – care play a crucial role in maintaining health and raising awareness about the use of carcinogenic substances, as a higher level of knowledge about oral cancer is observed among patients with higher educational levels and those who engage in regular physical exercise.^16, 28, 29^ This may explain lower educational level and lack of regular physical exercise as independent factors for the high – risk group for oral cancer.^30^

Patients with access to dental care have greater knowledge about the disease compared to those without access to services.^31^ Individuals at high risk for oral cancer often lack access to routine dental care^32^, as patients who have never visited a dentist or have not done so in over three years were more likely to be the high – risk group. This finding is concerning, as oral cancer is highest among the elderly population in Brazil; however, since edentulism remains common in this age group, individuals may consider dental visits unnecessary, further reducing the chances of early diagnosis and, more importantly, of receiving proper guidance and education from the dentist regarding lifestyle and habit modifications. Thus, the profile of the screened population may still be one of the reasons for the underreporting of oral cancer cases.^32^

Lower coverage of oral health care by primary health services and rural residents were also independent factors for the high – risk group for oral cancer, as observed in other studies.^33,34^ The lack of access to health services is one of the main factors related to the late diagnosis of oral cancer, often resulting in the need for more aggressive and disfiguring treatments, thereby reducing individual survival.^10^ In light of this, expanding oral health care coverage in primary and specialized care, prioritizing high – risk populations, is essential to improve the epidemiological scenario of oral cancer in Brazil.^33,35^ Similarly, implementing public policies to combat social inequalities, training oral health professionals, and expanding health education measures are crucial measures.^10^ Furthermore, the development of an individualized high – risk model for oral cancer targeted at the Brazilian population could be beneficial in case screening and in reducing the incidence and mortality of oral cancer, providing greater dignity and access to prevention and treatment.

The present study is important for including a representative sample of the country during the analyzed period, derived from national household – based surveys with a rigorous sampling design and a large sample size. The methodological rigor with which the data were collected (using validated instruments that ensure data reliability) is also noteworthy. Furthermore, this study established a definition of the high – risk profile for oral cancer in the Brazilian population. The main limitations are related to potential selection biases of the sample participants and measurement of the collected information, which were minimized by random sampling. For future research, it is recommended to conduct longitudinal studies with representative samples of population groups that further investigate relationships between less – studied risk factors, such as diet and sexual practices, in addition to country – specific studies to better define high – risk groups for oral cancer. This would allow for better planning and implementation of cancer prevention and control programs, particularly in developing countries and those with high oral cancer incidence rates.

## Conclusion

A large portion of the Brazilian population is exposed to risk factors for oral cancer and there are disparities in access to medical and dental health services and in the educational – preventive interventions of health professionals regarding risk factors for oral cancer. People of white ethnicity/race, low educational level, residing in rural areas, not registered in the ESF, who have never visited a dentist or did so more than 3 years ago, self – assessed their overall health as poor/very poor, and who do not engage in regular physical exercise are more likely to be in the high – risk group.

## Data Availability

The authors declare that all data generated or analyzed during this study are included in this published article.
